# Ultrasound evaluation of the abductor hallucis muscle: Reliability study

**DOI:** 10.1186/1757-1146-1-12

**Published:** 2008-09-25

**Authors:** Alyse FM Cameron, Keith Rome, Wayne A Hing

**Affiliations:** 1 AUT University, School of Rehabilitation & Occupation Studies, Health & Rehabilitation Research Centre, Private Bag 92006, Auckland, 1142, New Zealand

## Abstract

**Background:**

The Abductor hallucis muscle (AbdH) plays an integral role during gait and is often affected in pathological foot conditions. The aim of this study was to evaluate the within and between-session intra-tester reliability using diagnostic ultrasound of the dorso-plantar thickness, medio-lateral width and cross-sectional area, of the AbdH in asymptomatic adults.

**Methods:**

The AbdH muscles of thirty asymptomatic subjects were imaged and then measured using a Philips HD11 Ultrasound machine. Interclass correlation coefficients (ICC) with 95% confidence intervals (CI) were used to calculate both within and between session intra-tester reliability.

**Results:**

The within-session reliability results demonstrated for dorso-plantar thickness an ICC of 0.97 (95% CI: 0.99–0.99); medio-lateral width an ICC: of 0.97 (95% CI: 0.92–0.97) and cross-sectional area an ICC of 0.98 (95% CI: 0.98–0.99). Between-session reliability results demonstrated for dorso-plantar thickness an ICC of 0.97 (95% CI: 0.95 to 0.98); medio-lateral width an ICC of 0.94 (95% CI 0.90 to 0.96) and for cross-sectional area an ICC of 0.79 (95% CI 0.65 to 0.88).

**Conclusion:**

Diagnostic ultrasound has the potential to be a reliable tool for evaluating the AbdH muscle in asymptomatic subjects. Subsequent studies may be conducted to provide a better understanding of the AbdH function in foot and ankle pathologies.

## Background

The intrinsic muscles of the foot work as a functional unit in order to dynamically stabilise and assist in the support of the medial longitudinal arch [1-3]. The abductor hallucis muscle (AbdH) is the most medial muscle in the first layer of intrinsic muscles of the plantar surface of the foot. The proximal attachment is from the medial process of the calcaneus tuberosity, and its distal attachment is the proximal phalanx with or without attachment onto the medial sesamoid bone, or with insertion exclusively at the medial sesamoid bone [4]. As the tendon lies beneath the transverse axis of the first metatarsal, AbdH performs abduction and plantar flexion of the first metatarsal phangeal joint [5], being active in the late stance and toe-off phases of gait [6], and is a dynamic stabiliser of the longitudinal arch [7]. Musculoskeletal conditions such as hallux valgus (commonly known as a bunion) and pes planus can result in the structure and function of AbdH being adversely affected [5,8].

An observation commonly seen in patients with diabetes is atrophy of the intrinsic foot muscles, including AbdH, secondary to peripheral motor neuropathy [9]. Atrophy of the intrinsic foot muscle, which is a close representative of the level of motor dysfunction, is understood to result in an imbalance and altered arrangement, thereby causing prominent metatarsal heads, clawing of the toes, and the development of pressure areas predisposing to possible foot ulceration [9,10]. Alternatively, previous studies have also suggested that the muscle's anatomical line is altered and the strength of the muscle is compromised, consequently affecting the biomechanics of gait, the medial longitudinal arch configuration, and degenerative pes planus [5,8-11].

In hallux valgus the AbdH muscle is at a mechanical disadvantage as the distance between the proximal and distal attachments is increased, resulting in the muscle losing its abduction force [8,12]. An imbalance between the muscles of AbdH and Adductor hallucis muscle, which are responsible for coordinating the first metatarsophalangeal joint movements, is also evident, possibly leading to joint deformity [13]. These effects have been demonstrated to increase the load on the posterior tibial muscle, further increasing the likelihood of dysfunction [13].

There are a number of non-invasive techniques to image soft tissue structures. These include magnetic resonance imaging (MRI), computerized tomography (CT), and ultrasound (US), although not all are feasible or practical to operate in the clinical environment. Electromyography (EMG) has also been utilised to measure skeletal muscle activity [14]. Current evidence suggests a good correlation between ultrasound imaging and the "gold standard" of MRI and CT [15]. Furthermore, muscle imaging techniques such as MRI and US have been shown to be of value in inflammatory myopathies [15]. Ultrasound imaging is safe, non-invasive, easily performed and is a considerably less expensive process to undertake, all making it an advantageous piece of clinical equipment [16]. Ultrasonography has also already been shown to be a valid and reliable tool diagnostically in the imaging of skeletal muscle, producing quantitative and qualitative information about muscle architecture [17].

Ultrasound imaging has previously been used for measuring and analysing muscle cross-sectional area of vastus lateralis [18], lumber multifidus [19], and a range of intrinsic foot muscles that includes extensor digitorum brevis, the first interosseus dorsalis muscle, adductor hallucis and the first lumbrical muscle [11]. Ultrasound imaging has previously been used on the foot to measure plantar fascia band thickness in symptomatic and asymptomatic feet and to establish a plantar fasica index [20]. Methodologically, previous studies have used anatomical landmarks as reference points for the perpendicular position of the transducer in relation to the long axis of the limb, in a set repeatable patient position for carrying out the imaging of the identified muscle [11,19-21]. Quantitative analyses of the intrinsic foot muscles, including AbdH, have predominantly been performed on cadaveric feet through dissection [2,22]; however, to date there appears to be no studies that have measured AbdH in the asymptomatic population using ultrasonography. This may be beneficial for the diagnosis of pathology, monitoring adaptations, and providing evidence for the effectiveness of non-surgical interventions in relation to the AbdH muscle.

The aim of this study was to evaluate intra-tester within and between-session reliability using diagnostic ultrasound imaging of the AbdH dorso-plantar thickness, medio-lateral width and cross-sectional area.

## Methods

### Subjects

A convenience sample of thirty subjects were recruited from the University population. Subjects were included if they reported no history of inflammatory arthritis, previous foot or ankle surgery, diabetes, lower limb amputation, or severe hallux valgus as defined by the Manchester Scale [23]. All subjects provided written informed consent. The procedures used in this study were approved by the Universities Ethics Committee.

### Equipment

A Philips HD11 Ultrasound machine linear probe (L12-5 MHz, 50 mm broadband linear array) was used to scan images of the AbdH muscle. An Aquaflex^® ^Ultrasound Gel Pad (Fairfield, USA) was applied directly on the skin superficial to the AbdH muscle for optimal transducer contact and signal penetration. A stable platform held the foot in neutral position at zero degrees. Philips Q-lab Software (Release 5.0) was employed for data quantification.

### Experimental procedure

Subjects were laid in a supine position. The heel and plantar aspect, excluding the first metatarsal, of the involved foot rested against a stable platform designed to fix the ankle in a zero degree neutral position. The posterior aspect of the knee was supported in approximately 15 degrees flexion. The uninvolved leg was also supported.

**Figure 1:**
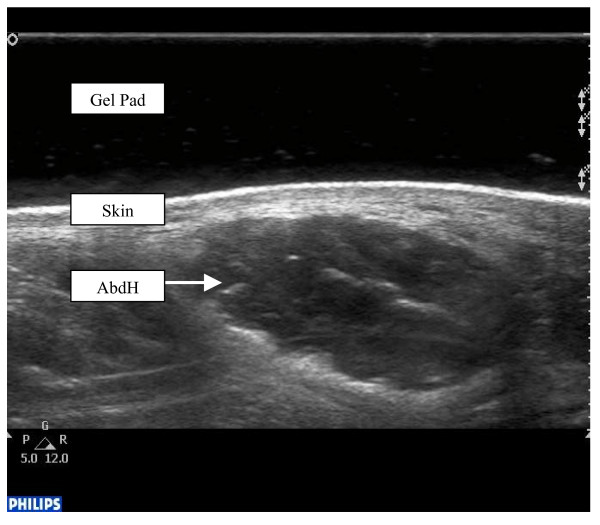
Ultrasound image of abductor hallucis muscle.

The researcher (AC) palpated the bony anatomical landmark of the anterior aspect of the medial malleolus and a perpendicular scanning line was drawn directly inferiorly. The ultrasound gel pad was applied onto the AbdH muscle belly, inferior to the medial malleolus. Scanning occurred with the transducer applied at a perpendicular angle to the aforementioned scanning line and long axis of the foot on the proximal aspect of the reference line to encompass the muscle fibres of AbdH. Minimal pressure was applied with the transducer to reduce any possible alterations to the muscle architecture (Figure 1).

**Figure 2:**
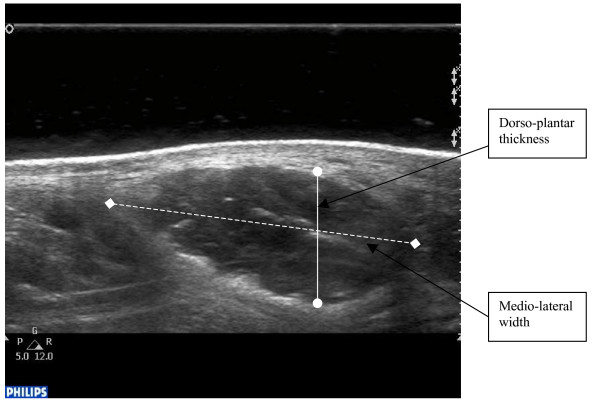
Abductor hallucis muscle with dorso-plantar thickness and medio-lateral width points marked.

**Figure 3:**
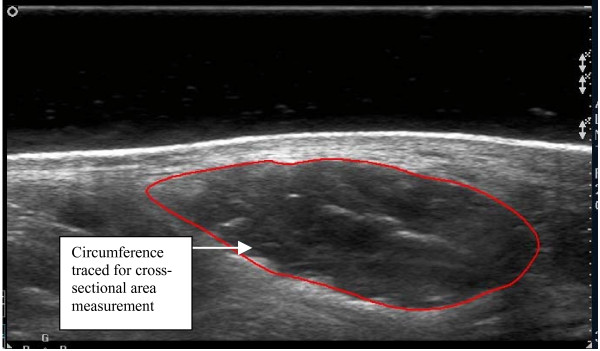
Abductor hallucis muscle with cross-sectional area outlined.

Using digital callipers, the dorso-plantar thickness and medial-lateral width of the AbdH was measured from the echogenic tissue interface between the muscle belly and the muscle fascia (Figure 2). The cross-sectional area measurement of the AbdH muscle was obtained through manual tracing of the muscle borders using the Philips Q-lab Software digital trace with edged detection capabilities (Figure 3).

The left and right foot AbdH muscle were scanned for digital investigation, and three separate repetitions of each foot were recorded in order to attain a mean measurement for each subject. The paired data was collapsed into a single measure by taking the mean of the left and right feet. The probe was reset in its holding port between each scan. This entire process was then repeated three to seven days later to gain between day test results. All ultrasonic imaging measurements were undertaken by AC who was a novice researcher but with training using US imaging over 3-months in a musculoskeletal paper run at the university. Additional training in scanning was undertaken prior to data collection by an experienced radiologist and sonographer based from the clinical scanning unit at the University Scanning Unit. In addition, one-to-one training sessions were undertaken with an experienced researcher in musculoskeletal US, Analysis was undertaken retrospectively and at the time of scanning to ensure blinding of the results. All images of the AbdH muscle captured were stored on the hard drive for later analysis.

### Data analysis

The baseline descriptive information from each subject was obtained. An analysis of the reliability of muscle cross-sectional area, medio-lateral width and dorsal-plantar thickness was conducted out using SPSS (version 15, SPSS Inc., Chicago, IL) Repeated measures (test-retest) reliability analyses utilised interclass correlation coefficients (ICC, 3.1) and 95% confidence intervals were obtained. As with other reliability coefficients, there is no standard acceptable level of reliability using the ICC [24]. It is stated that any measure should have an ICC of at least 0.6 to be useful [25]. Bland-Altman plots have been used to provide graphical representation of some of the key reliability findings [26,27]. The Bland-Altman method calculates the range within which the difference between the two occasions will lie with a probability of 95% [26,27].

## Results

Thirty subjects (20 female and 10 male) completed the study with a mean age of 28.24 ± 10.15 years, mean weight of 68.8 ± 12.35 Kg, and a mean height of 1.71 ± 0.97 m. Descriptive information of the AbdH muscle medio-lateral width, dorso-plantar thickness and cross-sectional area are presented in Table 1.

**Table 1:** Descriptive statistics of abductor hallucis muscle parameters.

Parameter	Day	Mean ± SD	ICC (95% CI)
Dorso-Plantar Thickness (mm)	1	11.55 ± 1.09	0.97 (0.98–0.99)
	2	11.52 ± 1.03	0.97 (0.98–0.99)
Medio-lateral Width (mm)	1	28.98 ± 2.69	0.97 (0.95–0.98)
	2	29.03 ± 2.54	0.97 (0.95–0.98)
Cross-sectional Area (mm^2^)	1	269.23 ± 35.47	0.98 (0.96–0.99)
	2	276.55 ± 33.98	0.95 (0.92–0.97)

**Figure 4:**
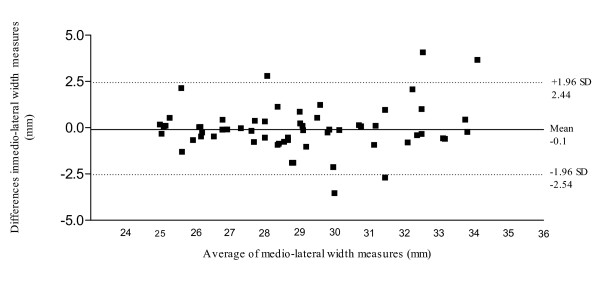
95% Limits of agreement for the measurement of abductor hallucis medio-lateral width (mm).

With respect to within-session reliability the results demonstrated high reliability for all three parameters measured (Table 2). Based on an average of the three repetitions, between-session reliability (Table 3) showed high agreement of measuring the dorso-plantar thickness of AbdH (ICC: 0.97; 95% CI: 0.95 to 0.98). High reliability was evident for medio-lateral width measurements (ICC: 0.94; 95% CI 0.90 to 0.96). Cross-sectional area of the AbdH was deemed as acceptable (ICC 0.79; 95% CI 0.65 to 0.88). Figure 4 illustrates the Bland & Altman plot between Session 1 and Session 2 for AbdH medio-lateral width, with a 95% limits of agreement, bias of -0.05, with SD of bias of 1.27 (Lower limit -2.54, Upper limit 2.44).

**Table 2:** Intratester within session reliability ICC values.

Parameter	Day	ICC (95% CI)
Dorso-Plantar Thickness (mm)	1	0.97 (0.98–0.99)
	2	0.97 (0.98–0.99)
Medio-lateral Width (mm)	1	0.97 (0.95–0.98)
	2	0.97 (0.95–0.98)
Cross-sectional Area (mm^2^)	1	0.98 (0.96–0.99)
	2	0.95 (0.92–0.97)

**Table 3:** Intratester between-session reliability ICC values.

Parameter	ICC (95% CI)
Dorso-Plantar Thickness (mm)	0.97 (0.95–0.98)
Medio-lateral Width (mm)	0.94 (0.90–0.96)
Cross-sectional Area (mm^2^)	0.79 (0.65–0.88)

**Figure 5:**
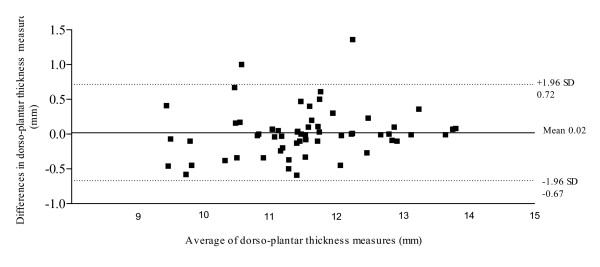
95% Limits of agreement for the measurement of dorso-planter thickness (mm).

**Figure 6:**
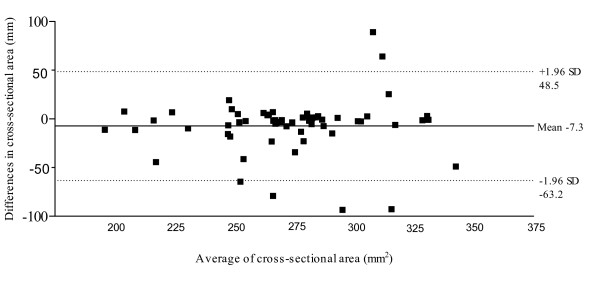
95% Limits of agreement for the measurement of cross-sectional area (mm^2^).

Figure 5 illustrates the Bland & Altman plot between Session 1 and Session 2 for AbdH dorso-plantar thickness, displaying a 95% limits of agreement, bias of -0.024, with SD of bias of 0.35 (Lower limit -0.67, Upper limit 0.72). Figure 6 illustrates the Bland & Altman plot between Session 1 and Session 2 for AbdH cross-sectional area, with a 95% limits of agreement, bias of -7.3, with SD of bias of 28.50 (Lower limit -63.18, Upper limit 48.54).

## Discussion

With any measuring system there needs to be of proven reliability and validity before being applied in a clinical setting, so that clinicians maybe assured of reproducible and meaningful results. Evaluating the reliability of muscle parameters has been in the past difficult. Only with an increase in accessibility to the higher-end US machines and also the development and increase in availability of low-cost musculoskeletal US machines has it been possible to conduct good reliability studies.

There is limited research exploring the AbdH muscle characteristics. There are however, previous studies utilising US, which have demonstrated that it is a statistically valid and reliable method for assessing the cross-sectional area of skeletal muscle [11,18]. A study by Reeves et al (2007) [18] observed measuring the cross-sectional area of vastus lateralis using US, comparing results to that of the findings from MRI (Table 4). Also a previous study used US to determine the cross-sectional area of extensor digitorum brevis, which again proved to be a reliable method of measurement (Table 4) [11]. Few studies to date have looked at measuring the muscle parameters of width and thickness using US imaging. An earlier study completed a within-session intra-tester and inter-tester reliability study to measure the thickness of multifidus muscle using US, which concluded in a very high inter-rater agreement of the thickness across both assessors (Table 4), therefore indicating that the aforementioned parameters can be measured reliably [19]. The current study's methodology using US was developed based on the protocols of previous work. These included the utilisation of anatomical landmarks as reference points, allowing time for muscle fluid shifts to occur before scanning, the perpendicular transducer angle, and neutral testing position of the ankle [20,26,28].

**Table 4:** Comparison of results gained from other ultrasound studies measuring muscle parameters.

Study	Parameter	Results
Severinsen & Andersen (2007) ^25^	Cross sectional area: extensor digitorum brevis	*r *= 0.75
Reeves et al (2004) ^21^	Cross sectional area: vastus lateralis	ICC = 0.99
Wallwork et al (2007) ^29^	Width: L2–L3 Multifidus	ICC = 0.96
	Width: L4–L5 Multifidus	ICC = 0.97

From a clinical perspective, the role of the AbdH muscle is still yet to be determined but previous work suggests that the AbdH muscle and its distal attachment play an important role in the aetiology as well as in therapy of hallux valgus [5,29,30]. In orthopaedic, plastic and reconstructive surgery the AbdH muscle allows for rising interest as it is taken as a graft for flap-surgery [5]. Hypertrophy of the AbdH muscle have been reported to be an aetiological factor in tarsal tunnel syndrome [31]. Myofascial syndrome of AbdH muscle has been reported to cause heel pain [32] and acupuncture meridians utilising the muscle belly of AbdH muscle has also been reported in the literature [33]. However, the previous studies on evaluating the muscle parameters of the AbdH muscle has been limited by questions related to the reliability, validity, standardisation, methodology, and the ability to detect changes over time. The current study, by assessing the within and between session reliability of image acquisition of the AbdH muscle using a standardised methodology to measure medio-lateral width, dorso-plantar thickness and cross-sectional area demonstrated high intra-tester reliability.

Limitations to the current study included measurement error in evaluating the cross-sectional area of AbdH through manual digital trace. Future digital/computer generated mapping of the muscle cross-sectional is a possibility; Reeves et al (2004) [18] reported that reducing measurement error could be undertaken by comparing US cross-sectional results to that taken from an MRI in order to assure the accuracy of the cross-sectional area. However, this is a costly method to adopt in the clinical setting. In the current study, the ultrasonographer was not blinded to the identity of the subjects examined, but randomising the sequence and subjects reduced the potential for bias. Future studies may consider blinding the ultrasonographer to reduce measurement error. Inter-tester reliability was not assessed in the current study but is being planned for future work. A further limitation is the issue of obtaining the spatial relationship of irregular anatomical structure such as the AbdH muscle using 2D sonography. Future work may take into account 3D measurements in conjunction with new technology. Utilising 3D US transducers are planned for future research using the Philips U22 which has 3/4D capabilities. Previous work on multifidus reported on the muscle activity using EMG simultaneously with measuring and monitoring the muscle with US [34]. Future work could utilise the current standard method in conjunction with EMG to evaluate functional parameters of AbdH muscle in conditions such as hallux valgus and tarsal tunnel syndrome.

## Conclusion

Using US in the current study baseline results have been reported for intra-tester reliability in the measurement of the AbdH muscle. Future studies using the current protocol may give a clearer understanding of the role the AbdH muscle plays in pathological conditions that may impact on the foot and ankle.

## Competing interests

The authors declare that they have no competing interests.

## Authors' contributions

AC carried out the literature review, piloting, data collection and drafted the manuscript. KR and WH participated in the design of the study, statistical analysis and drafting of the manuscript. All authors read and approved the final manuscript.
